# Polyomaviruses detectable in head and neck carcinomas

**DOI:** 10.18632/oncotarget.25202

**Published:** 2018-04-27

**Authors:** Leonard Poluschkin, Jaana Rautava, Aaro Turunen, Yilin Wang, Klaus Hedman, Kari Syrjänen, Reidar Grenman, Stina Syrjänen

**Affiliations:** ^1^ Department of Oral Pathology and Oral Radiology, Institute of Dentistry, Faculty of Medicine, University of Turku, 20520 Turku, Finland; ^2^ Department of Pathology, Turku University Hospital, 20521 Turku, Finland; ^3^ Department of Virology, University of Helsinki, and Helsinki University Hospital, 00290 Helsinki, Finland; ^4^ Department of Clinical Research, Biohit Oyj, 00880 Helsinki, Finland; ^5^ Department of Otorhinolaryngology – Head and Neck Surgery, University of Turku and Turku University Hospital, 20520 Turku, Finland

**Keywords:** polyomaviruses, SV40, JCV, BKV, head and neck cancer

## Abstract

Polyomaviruses (PyV) independent or jointly with human papillomavirus (HPV), might have a role in head and neck carcinomas (HNSCC). We analyzed the prevalence and viral DNA loads of SV40, JCV and BKV with quantitative PCR (qPCR) and all 13 HPyVs with a novel Multiplex method in 82 HNSCC samples with known HPV status and disease-specific survival (DSS) and 24 HNSCC cell lines.

JCV was the most prevalent PyV present in 37% of HNSCC and the most prevalent sites were lip (80%), larynx (67%) and oral cavity (59%). JCV viral load was highest in larynx but variation was wide (152514 mean copies/μg DNA, SD± 304820). BKV was found only in one oral carcinoma with low viral load. SV40 was detected in 60% lip and 20.7% oral carcinomas with low copy numbers (6.6- 23.7 copies/μg DNA). Altogether, 86% of JCV-positive samples were co-infected with HPV (p=0.001), with no impact on DSS. Agreement between qPCR and Multiplex methods was substantial (Cohen's kappa= 0.659). Multiplex method detected additional HPyV in five samples. JCV was found in 9/24 HNSCC cell lines, all deriving from oral cavity. Our data provide evidence that JCV might have a role in HNSCC as independent virus or co-factor of HPV.

## INTRODUCTION

Head and neck cancers (HNSCCs) comprise a heterogeneous group of tumors including cancers of the lip, oral cavity, nasal cavity, paranasal sinuses, nasopharynx, oropharynx, hypopharynx, larynx and salivary glands. Nearly 90% of these malignancies are squamous cell carcinomas (SCC). HNSCCs represent a major global cancer burden, with over 686.000 annual new cases. The age-standardized mortality rate was 4.9/100.000 in 2013 [1].

Nearly 80% of the HNSCCs are ascribed to smoking and heavy alcohol use [2]. The first evidence of human papilloma virus (HPV) infection in head and neck carcinogenesis was provided already 35 years ago [3]. During the past 15 years, the research on HPV in HNSCC has been overwhelming, confirming that a subgroup of HNSCC is caused by HPV, especially HPV16. The HPV-association is strongest for oropharyngeal cancers, yet highly variable worldwide [4]. Recently, the role of other viruses, e.g. HSV or EBV as co-factors for HPV-induced carcinogenesis has gained increasing interest, as discussed in a recent review [5].

Polyomaviruses (PyVs) resemble papillomaviruses in structural and functional properties, being small, non-enveloped, double-stranded, circular DNA viruses. The polyoma- and papillomaviruses were originally classified into the Papovaviridae family, which was split into the two families, papillomaviridea and polyomaviridae only in 2004 [6]. Up to now, 13 human polyomaviruses (HPyVs) have been identified. BKV was the first and JCV the second in this group, identified in 1971 [7, 8]. Primary infections with BKPyV occur mostly in childhood before the age of 7 years while the timing of primary JCV infection tends to be more common during the second and third decades of life, and are presumed to be subclinical with periodical virus shedding to urine and saliva [9–11]. While PyV-mediated diseases are known to occur mostly in immunocompromised patients, their role in human carcinogenesis – except for Merkel cell virus (MCPyV) [12] – is still controversial, and systematic studies on all the HPyVs known, with regard to cancers, are scanty (13-14).

BKPyV and JCPyV belong to the genus Betapolyomaviruses, together with the simian vacuolating virus 40 (SV40), discovered in 1960. SV40, one of the first tumor viruses described, might also circulate among humans although it is a non-human primate polyomavirus. It is estimated that over 100 million people may have been exposed to SV40 due to the contaminated polio vaccine administered during 1955–1963. Furthermore, it has been shown that polio vaccines produced from early 1960s to about 1978 from a major eastern European manufacturer were also SV-40 contaminated and widely used throughout the world [15]. The virus originated from the monkey kidney cell cultures used to produce the polio vaccine [16]. Shah and Nathanson 1976 [17] reported that intranasal administration of “old SV40 contaminated polio vaccine, SV40 produced a low grade infection with virus shedding in the respiratory tract and a low level antibody response in a few of the volunteers. Accordingly, several inhabitants in Finland also might have also been exposed to SV40 via this route in the late 1950's until 1964. The role of SV40 in human carcinogenesis has been widely discussed but the results have been conflicting. Shah in his mini review on SV40 and human cancer concluded that the most recent evidence does not support the notion that SV40 contributed to the development of human cancers [18]. SV40 has been shown to act as a coactivator of asbestos in mesothelial oncogenesis [19] although the detection rates of SV40 in mesothelioma show considerable variability. Thus, causal relationship between SV40 and this tumor cannot be drawn, yet [20].

The main oncogenic viral protein of SV40, JCPyV and BKPyV is the large tumor antigen (T-Ag) that is able to bind several host proteins, including retinoblastoma (pRb) and p53. This will result in dysregulation of the cell cycling machinery as with the high-risk HPVs [13, 14]. Thus, SV40, JCPyV and BKPyV might be potential co-factors in HPV-induced carcinogenesis in the head and neck region. Both JCPyV and BKPyV can be detected in saliva even if their reservoir is unknown. There is evidence that tonsils, spleen, and lymph nodes are permissive to JCV/BKV, indicating that those tissues might have a role in viral persistence [10, 21, 22]. There are few reports on these viruses in HNSCCs but the numbers of cases analyzed have been limited and the results are conflicting [23–26].

The present study had three main aims; 1) to analyze the presence and SV40, JCV and BKV DNAs in HNSCCs and their impact on disease-specific survival (DSS), 2) to analyze the role of SV40, JCV and BKV as co-factors for HPV and some other herpesviruses previously analyzed in this same cohort, and 3) to study the presence of SV40, JCV and BKV DNAs in cell lines derived from HNSCCs. Moreover, we were interested in comparing the qPCR-based method for SV40, JCV and BKV DNA detection with a new multiplex method capable of detecting all 13 HPyVs known.

## RESULTS

### HNSCCs

The presence of SV40, JCV and BKV in HNSCCs (n=82) was first analyzed with qPCR, all three viruses in separate qPCR reactions. Table 1 summarizes the prevalence of SV40, JCV and BKV as well as their copy numbers in the HNSCC samples, stratified by their anatomic sites. The most prevalent polyomavirus by far was JCV, found in 37% of the 81 HNSCCs (one sample missing due to lack of DNA). The carcinoma site most frequently associated with JCV was the lip (80%), followed by the larynx (66.7%), the oral cavity (58.6%), the nasopharynx (33.3%) and the hypopharynx (25%). The copy numbers were highest in the larynx, yet varied widely among cancer sites (152514 mean copies/μg DNA, SD± 304820). The highest copy number was in a laryngeal sample, 609,756 copies/μg DNA pointing to an active viral replication. Overall, the average copy number levels in lip and oral cavity carcinomas were very similar (over 600 copies/μg DNA), but were markedly lower in nasopharyngeal and hypopharyngeal carcinomas; 156.8 and 122.3 copies/μg DNA, respectively. BKV was found in only one of the 29 oral cavity carcinomas, with 32.3 copies/μg DNA. SV40 DNA was found in three of the five lip carcinomas (60%), and in 6/29 oral cavity carcinomas (20.7%). None of the pharyngeal samples tested SV40 positive, but two of the laryngeal carcinomas were positive (28.6%). The copy numbers of SV40 in all anatomic sites were low, the means ranging from 6.6 to 23.7 copies/μg DNA with an overall mean copies being only 12.4copies/ug. The copy numbers can be expressed also as copies per 1000 cells by estimating the DNA content of one diploid human cell to be 6 ng meaning that there are only 74.4 copies of SV40 in 1000 cells. Interestingly, SV40, JCV or BKV were not found in any of the oropharyngeal carcinomas.

**Table 1:** Prevalence of viral DNA of SV40, JCV and BKV and their DNA loads according to anatomical subsites of HNSCCs

Location	SV40+ %	SV40 copy numbers per μg DNA (Mean +SD)	JCV+ %	JCV copy numbers per μg DNA (Mean +SD)	BKV+ %	BKV copy numbers per μg DNA (Mean +SD)
Lip	3/5 60.0%	6.63 (2.76)	4/5 80.0%	702.23 (1120.01)	0/5 0%	-
Oral Cavity	6/29 20.7%	11.52 (10.26)	17/29 58.6%	675.09 (1126.14)	1/29 3.4%	32.27
Oropharynx	0/23 0%	-	0/23 0%	-	0/23 0%	-
Nasopharynx	0/6 0%	-	2/6 33.3%	156.81 (183.75)	0/6 0%	-
Hypopharynx	0/12 0%	-	3/12 25.0%	122.31 (103.23)	0/11 0%	-
Larynx	2/7 28.6%	23.74 (29.23)	4/6 66.7%	152514.03 (304820.00)	0/6 0%	-
**Total positivity and overall mean copy numbers per μg DNA**	**11/71** **13.4%**	**12.42** **(13.25)**	**30/81** **37.0%**	**20834.08** **(111233.62)**	**1/81** **1.2%**	**32.27**

### Gender and HPyVs

Women were significantly older than men at the time of diagnosis (p=0.026), with mean ages of 66.9 and 59.9 years, respectively. There was no gender association with JCV or SV40 detection. SV40 positive patients were slightly older than the negative ones, without statistical significance. JCV-positive patients were significantly older than JCV-negative patients (66.7 years vs. 59.3 years, p=0.017).

### HNSCC survival

**Figure 1:**
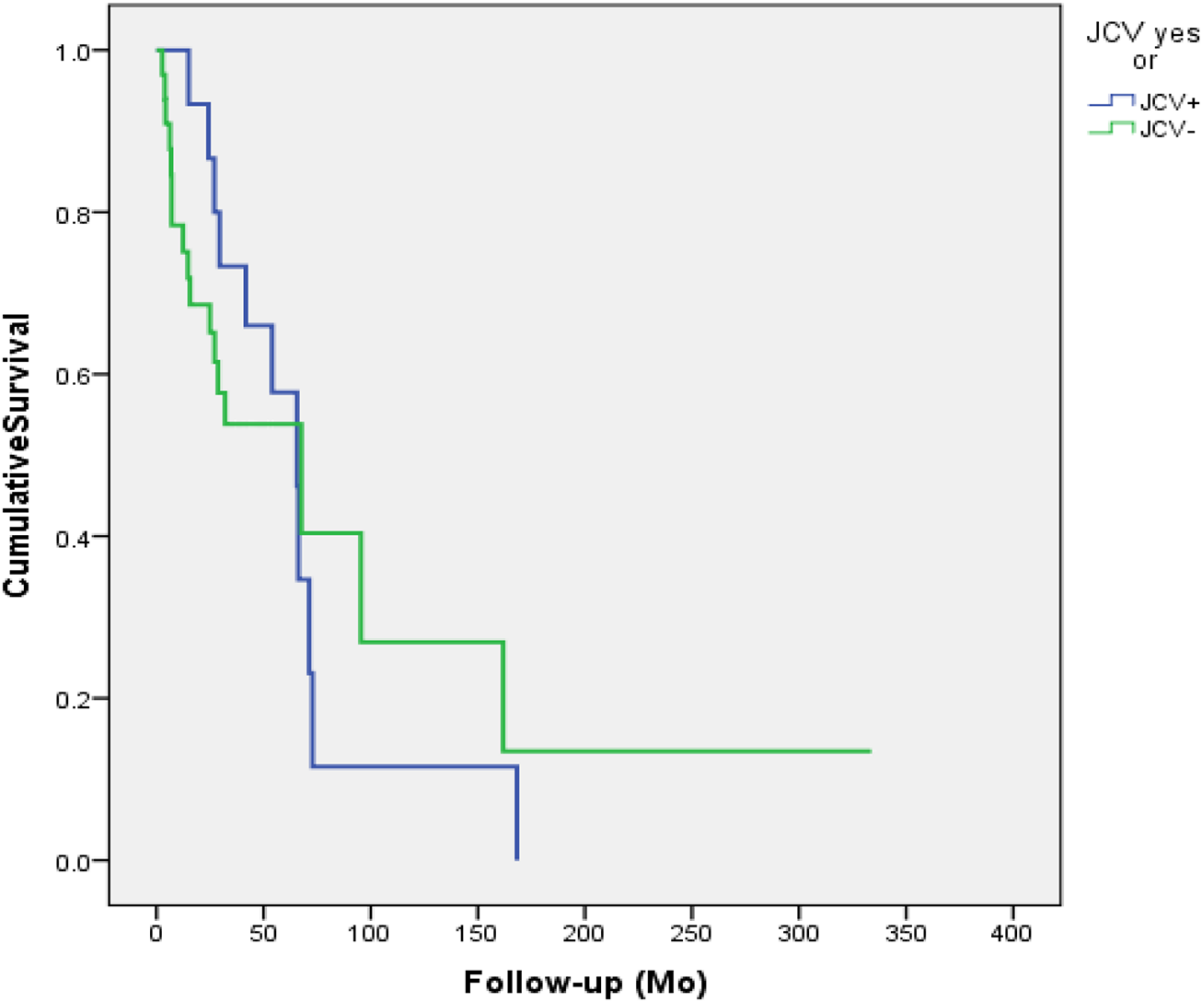
Disease specific survival of the patients accroding to the JCV status in the cancer tissue JCV had no efect on the outcome of the patient (p=0.914).

**Figure 2:**
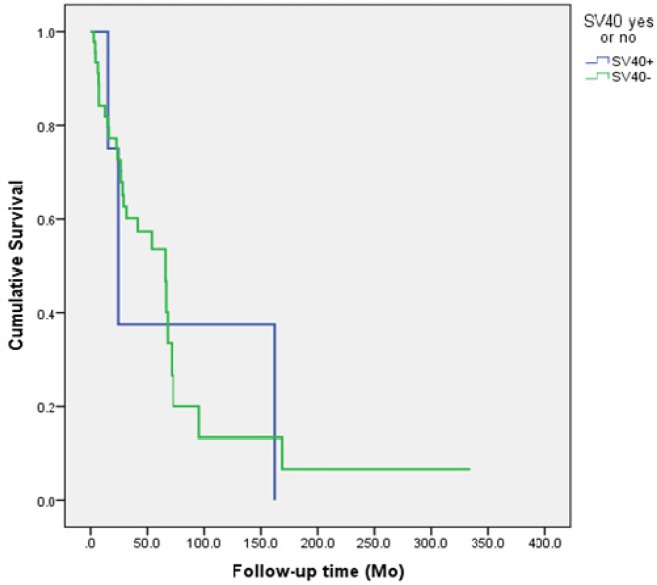
The survival of the patients according to the SV40 status of the head and neck cancer The presence of SV40 had no effect on the patient's survival (p=0.894).

The outcomes of the patients with JCV and SV40-infected tumors are summarized in Figures 1 and 2. Neither SV40 nor JCV positivity showed any association with the patient's disease specific survival (DSS). There were no statistically significant differences in the copy numbers of SV40 or JCV according to the treatment mode (surgery or surgery and irradiation), TNM-classification or disease stage (I-IVA). However, the copies of SV40 were significantly lower in the patients without any metastasis to regional lymph nodes. None of the cancer patients had N2, while 4 of 11 patients had N1 disease with SV40 copies of 22.7/μg DNA. Seven of 11 SV40 positive patients had N0 with mean copy numbers of 6.5/μg (p=0.042).

### Co-infections with HPV or/and HSV-1

Co-infections of polyomaviruses with HPV and/or HSV-1 are presented according to age and gender in Table 2. JCV infection was associated with HPV infection as 86.2% of the JCV positive HNSCC samples were also HPV positive (p=0.001). HPV was also present in 70% of the SV40 positive samples, but there was no statistically significant difference in HPV prevalence among the SV40 positive or negative samples. Only one sample was BKV positive and it also had a co-infection with both HPV and HSV-1. The co-infections of HPyV with HPV or HSV-1 alone or together did not affect the patient's DSS.

**Table 2:** Prevalence of JCV, SV40 and BKV according to patient age and gender and co-infections with HPV and/or HSV-1

	JCV (N=81)		SV40		BKV	
	JCV+ (N=30)	JCV- (N=51)	SV40+ (N=11)	SV40- (N=71)	BKV+ (N=1)	BKV- (N=80)
Mean age (years)	66.7	59.3	65.9	61.7	76	61.9
Gender men women	17/53(32.1%) 13/28(46.4%)	36/53(67.9%) 15/28(53.6%)	6/54 (11.1%) 5/28 (17.9%)		0/53 1/28	53/53 27/28
HPV+^*^ HPV-	25/29(86.2%) 4/29(13.8%)	25/51(49.0%) 26/51(51.0%)	7/10 (70.0%) 3/10 (30.0%)	43/71(60.6%) 28/71(39.4%)	1/1 0/1	
HSV-1+ HSV-1-	0/19 (0.0%) 19/19 (100%)	3/41 (7.3%) 38/41(92.7%)	0/7 (0.0%) 7/7 (100%)	3/54 (5.6%) 51/54(94.4%)	1/1 0/1	
HPV+ & HHV-1+	0/19 (0.0%)	3/41 (7.3%)	0/7 (0.0%)	3/54 (5.6%)	1/1	2 (3)

^*^The HPV DNA positivity was statistically significantly associated with the JCV positivity (p=0.001).

### UT-SCC cell lines

Altogether, 23 cell lines were analyzed with qPCR. Only JCV was present in nine cell lines; all established from oral cavity cancers (Table 3). Seven were established from tongue and two from gingival carcinoma. Of the patients, six were women and three were men. The histology of the original tumors of the nine JCV-positive cell lines was well differentiated SCC in five cases, moderately differentiated in three, and poorly differentiated in one. Three of the patients had presented with regional neck metastases, yet none with distant metastases. JCV copy numbers ranged from 213.8 to 444.2 copies per μg DNA with an average of 86.9 copies.

**Table 3:** Characteristics of the original tumors of the JCV DNA positive cell lines

Cell line	JCV copy numbers per μg DNA	Origin on the cell line	TNM	Grade	Gender	Age years
**UT-SCC-16A**	30.3	tongue	T3N0M0	G3	woman	77
UT-SCC-30	17.1	tongue	T3N1M0	G1	woman	77
UT-SCC-40	10.7	tongue	T3N0M0	G1	woman	65
UT-SCC-43A	35.8	gingiva mandible	T4N1M0	G2	woman	75
UT-SCC-55	19.3	gingiva mandible	T4N1M0	G2	man	76
**UT-SCC-81**	13.4	tongue	T2N0M0	G1	man	48
UT-SCC-92	17.7	tongue	T2N0M0	G1	woman	57
**UT-SCC-95**	19.9	tongue	T1N0M0	G1	woman	83
**UT-SCC-97**	64.2	tongue	T2N0M0	G2	man	67

Cell lines with bold letters tested JCV positive also with Multiplex test.

### Comparison of the qPCR and Multiplex methods

Comparison of the two methods was done by using the DNAs extracted from 42 HNSCC samples and 23 cell lines. Table 4 summarizes the concordance of the JCV results. Of the 42 tested HSCC samples 23 (54.8%) and 19 (45.2%) were JCV positive and negative with the Multiplex method, respectively. qPCR found JCV in 21 (91.3%) of the 23 Multiplex positive samples. In all, the results were concordantly JCV-negative in 74.7% of the cases (14/19). The overall concordance between the two methods in detecting JCV was statistically highly significant (p=0.0001) with a kappa value of 0.659.

**Table 4:** Comparison of the qPCR and Multiplex methods in detecting of JCV

	JCV detection with qPCR		JCV detection with Multiplex method		Total
			JCV+	JCV-	
**JCV yes or no**	**JCV+**	N	21	5	26
		% with JCV yes or no	80.8%	19.2%	100.0%
	**JCV-**	N	2	14	16
		% with JCV yes or no	12.5%	87.5%	100.0%
**Total**		N	23	19	42
		% with JCV yes or no	54.8%	45.2%	100.0%

With the Multiplex method, four HNSCC samples tested positive for MCPyV, and one sample for HPyV6. All the MCPyV positive samples were also JCV positive. Of these two were women and two men, with respective age ranges of 60-76 and 51-95 years. The carcinomas were located in the lip, maxillary sinus, and tongue (two). Interestingly, the woman with maxillary carcinoma had also been diagnosed with skin carcinoma of the upper lip at the age of 42 years. The HPyV6 positive sample was derived from a female with laryngeal and base of the tongue cancer.

### Cell lines

In the cell lines the Multiplex method gave positive results exclusively for JCV, as also did the qPCR. Of the nine qPCR-positive cell lines, four (UT-SCC-16A, UT-SCC-81, UT-SSC-95 and UT-SCC-97) tested positive with the Multiplex method (Table 3). These cell lines were all derived from the tongue carcinomas.

## DISCUSSION

This study confirmed that polyomaviruses are detectable in head and neck cancers. JCV was the most frequent (37%) in the HNSCCs, followed by SV40 in 13.4% and BKV in 1.2%. Only few previous studies are available providing evidence that carcinomas of the tongue, pharynx and larynx might be associated with PyVs [23, 25–27].

All oropharyngeal carcinomas remained PyV negative in our study unlike in that of Zheng and coworkers [27]. They reported significantly higher copy numbers in carcinomas of the mobile tongue and pharynx than in the respective healthy mucosa. Contradictory to oropharyngeal carcinomas, we were able to detect JCV DNA in 2/6 and 3/12 naso- and hypopharyngeal carcinomas, respectively. In our study, the most prevalent sites for JCV and SV40-associated HNSCC, independently, were larynx followed by lip and oral cavity. According to Zheng and coworkers [27], there was no significant difference in the prevalence of JCV DNA in normal laryngeal mucosa and in laryngeal carcinoma.

Kutsuna et al. [23] reported a connection between high JCV viral load and tongue carcinomas, in line with our observations. They demonstrated JCV viral loads of more than 200 copies/μg in 36 of the 39 tongue carcinoma samples analyzed. Of these, 15 contained more than 1000 copies/μg DNA. Accordingly, they concluded that JCV might be a risk factor for tongue carcinogenesis. Our data do support this hypothesis, with JCV DNA demonstrated in 58.6% (17/29) of oral cavity carcinomas, even the copy numbers we found were lower. More importantly, we showed that of the JCV-positive cell lines derived originally from oral cavity carcinomas, seven originated actually from tongue. This indicates that tongue might have a particular predilection for transforming JCV infections. The copy numbers in oral carcinoma samples (675 mean copies/μg DNA) were higher than in the cell lines (25 copies/μg DNA). Unfortunately, we did not have any of the original tumors of these cell lines available for polyomavirus testing. However, based on our previous studies on HPV, it was shown that viral genome can disappear in the early passages of cell cultivation [28]. This also supports the concept of hetero-population and cell selection, in line with the hit and run hypothesis.

The JCV copy numbers especially in laryngeal carcinomas were high. However, the number of cases analyzed was small and the ranges in copy numbers high. These observations warrant further studies on the association of JCV in laryngeal carcinogenesis, even if none of the laryngeal cancer cell lines tested JCV positive.

Nearly 14% of the HNSCC samples studied contained SV40 DNA, but the copy numbers were low. Contradictory to our results, Palmieri and coworkers [29] did not find any SV40 in oral samples derived from either healthy tissue or cancer. SV40 is not originally hosted by humans but by monkeys and it has entered the human population mainly via contaminated polio vaccines in the 1950's and early 1960's [16, 30]. According the review by Shah (18) SV40 seroreactivity in cancer cases and controls varied from 2.5% to 15.2% and from 2.9% to10.5%, respectively. This indicates that immunoreactivity to SV40 is found but the frequency of seropositivity is nearly the same in the cases and the controls, similarly as recently reported by Antonsson et al. [31] even the seroprevalence of BKV, JCV and SV40 in the controls, they found, was much higher, 89%, 48% and 21%, respectively. A recent study showed SV40-specific serum antibodies detectable in pregnant women at the time of delivery and in cord blood; yet with no evidence of trans-placental transmission of SV40. These data suggest that SV40 is still circulating at low prevalence long after use of the contaminated vaccine. [32]. SV40 has also been found in tonsils of immunocompetent children. It seems likely that the oropharyngeal lymphatics may be able to host DNA virus persistence [10, 24, 33, 34]. Thus, SV40 known to be able to transform also oral keratinocytes advocates further studies on its role in head and neck carcinogenesis.

Both BKV and JCV are ubiquitous viruses, and up to 80% of the adult population is seropositive for both [35, 36]. Given that we did not have any biopsies from normal head and neck mucosa, one can always argue the specificity of our findings. Previous studies have shown that polyomaviruses, especially JCV and SV40, can infect B-cells and even establish a latency in these cells. Accordingly, oropharyngeal carcinomas would be the most likely samples to test polyomavirus-positive due to their abundance of B-cells. However, in the present analysis, all oropharyngeal cancers remained negative for JCV, BKV and SV40 providing indirect evidence against these viruses being present in lymphocytes of the tumor tissues.

In this context, a reference is made to the report of Palmier and coworkers [29], who studied 294 tumor samples (paraffin embedded) and 237 matched controls from oral cavity for the presence of BKV, JCV and SV40, using qPCR. They found only one BKV-positive tumor among all 483 samples including both the tumors and the controls. However, the viral load was very low; only 0.18 viral genome per cell. Furthermore, 15 tumor samples and 18 controls showed some PCR endpoint signals for the presence of BKV. These samples were also classified as negative as they did not reach the threshold of 1.0 viral genome per cell. If we estimate that there are nearly 100,000 carcinoma cells in one μg of DNA, only a few samples from the lip, oral cavity and laryngeal carcinomas would have been signed as JCV-positive in our study. However, we can argue this approach because our samples were examined also with a novel, somewhat less sensitive Multiplex method [37]. Altogether, 91% of the 23 samples testing JCV-positive with Multiplex were positive also with the qPCR. Furthermore, 74% of the multiplex-negative samples were also negative with the qPCR. Four of the cell lines tested positive with both methods. Thus, the overall concordance between the two methods was excellent indicating that the PyV findings achieved with qPCR correctly reflect the presence of viral DNA in the tumours and corresponding cell lines.

In the present series, the PyV-positive patients were slightly older than the virus-negative ones, yet the difference was significant only with JCV. Zeng et al. [27] did not find any effect of age on JCV viral loads. Polz and coworkers [25] showed an age association in BKV-positive oral cancer. Zheng et al. [27] also reported that JCV load was unrelated to tumor grade, consistent with our data. The same was reported by Polz et al. [25] with regard to oral cancer and BKV positivity.

In addition to their independent oncogenic role, also an auxiliary cancer-driving role of HPyV infections in combination with HPV infections deserves some consideration [13, 26]. SV40 was shown to increase the transforming activity of HPV16 by 10- to 15-fold *in vitro* [38]. A recent review showed that co-infections of HPyV and oncogenic HPVs are not infrequent [39]. This is particularly true in anal cancer and high-grade cervical intraepithelial lesions (CIN) [40]. In the present study, both SV40 and JCV were frequently found in concert with HPV. Interestingly, no particular HPV genotype preferentially escorted JCV. No previous studies exist on JCV/HPV or SV40/HPV co-infections in HNSCC. Comar and coworkers [32] reported JCV in 7% of the high-grade CIN but not in normal cervical samples. Alosaimi et al. [40] found 3-fold higher JCV frequency in cervical cancers than in normal cervical smears among HIV-positive women. This suggests a role of JCV in cervical cancer in HIV-co-infected, immunosuppressed patients. Polz-Gruszka [26] and coworkers found that 4.8% of the oropharyngeal cancers exhibited HPV/BKV co-infection. We found only one sample with HPV/BKV co-infection, not derived from oropharynx. The presence of JCV, BKV or SV40 either alone or with HPV-co-infection did not affect disease specific survival (DSS), as expected. Our cohort did not raise any oropharyngeal cancer type above others in predisposition to HPV/HPyV co-infection. HPV is known to be associated with a better survival, especially if the patient has never smoked or has no other comorbidity [41, 42].

When detecting polyomaviruses, JCV, BKV and SV40, with PCR or qPCR a remark has to make on the possibility of the crossreactivity due to the sequence homology. We have used qPCR using the method described by McNees et al. 2005 (43). Based on their carefully controlled study quantification of the target genes was sensitive and specific over a 7 log dynamic range. Ten copies each of the viral and cellular genes were reproducibly and accurately detected. The primers and probes used to detect the viral genes were specific for each virus and there was no cross reactivity within the dynamic range of the standard dilutions as we also showed in our experiments. The sensitivity of detection for these assays was not reduced in human cellular extracts. SV40 plasmid has never been available in our laboratory omitting the possibility of plasmid contamination which has been blamed for false positive results at low copy numbers.

To conclude, our results showed that JCV was found in HNSCC and also SV40 but the latter at very low copy numbers. Like BKV, both of these viruses have been implicated in human oncogenesis. Head and neck region is an important area to explore virus-related cancers. Epithelial cells of head and neck mucosa are continuously exposed to DNA-damaging agents, resulting in cellular responses that induce cell-cycle arrest or apoptosis to allow repair or elimination of the damaged cell. PyVs have several mechanisms to promote cellular proliferation and efficiently replicate their own DNA even in the presence of cellular stress. T-antigen is the main oncogenic protein of JCV, BKV and SV40. It can inactivate p53 and the members of pRb family, resulting in deregulation of cell cycle checkpoints and elimination of p53-mediated pro-apoptotic activity similarly as done by high-risk HPVs. Additionally, T-antigen exerts its oncogenic activity by deregulating the Wnt signaling pathway through stabilization of β-catenin and its interaction with the IGF-IR signaling system for cellular transformation. The Wnt/β-catenin signaling pathway has also been noted to be upregulated in HNSCC [44]. Thus, further studies are needed to understand the role of PyVs in HNSCC either as independent infections or co-infections with other oncogenic viruses like HPV and EBV.

## MATERIALS AND METHODS

### Patients and HNSCC samples

This study consists of 82 patients with primary HNSCCs. Of them 54 were men and 28 women with a mean age of 62.3 years (±SD 13.7 years) and a range from 27 to 96 years. All patients were treated with curative intent at Turku University Hospital between 1988-2015, where the management of all HNSCC patients is centralized from South-Western Finland, Ahvenanmaa and Satakunta. The tumor samples were obtained during the primary surgery (for temporary or permanent treatment) or as biopsies. The samples were freshly frozen in liquid nitrogen and stored at -70°C until use. Only samples histologically representing the original tumors were included in the study. Clinical data were collected retrospectively from the hospital records. The study has been approved by the Ethical Committee of the Hospital District of South-Western Finland (4/2009).

### Cell lines

Altogether, 23 UT-SCC (University of Turku squamous cell carcinoma) cell lines established from HNSCCs as described earlier were available for the present study [45]. The cells were cultured in Dulbecco's minimal essential medium (D-MEM, Paisley, UK), supplemented with 1% non-essential amino acids, 2 mM L-glutamine, 50 mg/ml streptomycin, 100 U/ml penicillin and 10% fetal calf serum, in 75 mm^3^ bottles. The cell lines were harvested at 80% confluence.

### DNA extraction

#### Biopsy samples

HPV DNA was extracted with the high salt method [46]. In brief, the samples were lysed in lysis buffer (10mMTris-HCl, 400mMNaCl and 2 mM EDTA, pH 8.2) with proteinase K. After digestion, proteins were precipitated with saturated NaCl and DNA with ethanol.

#### Cell lines

The cell pellet was suspended in 50 μl of extraction solution prepared by combining 0.5M EDTA pH 8.0, 1M Tris pH 8.0, Tween-20, Proteinase K at 20 mg/ml and ultrapure water. The tubes were incubated at 37°C overnight after which the proteinase K was inactivated by heating to 95°C for 10min. After digestion, proteins were precipitated with saturated NaCl and DNA with ethanol.

### HPV and HSV DNA detection

The presence of HPV and HSV-1, in these tumors has been reported earlier [47]. For HPV detection and genotyping, nested PCR (MY09/MY11 as external primers and GP05+/bioGP06+-as internal primers) and Luminex-based Multimetrix® kit (Progen Biotechnik GmbH, Heidelberg, Germany) was used. The kit detects 24 HPV genotypes (low risk types 6/11/42/43/44/70 and high risk types 16/18/30/31/33/35/39/45/51/52/53/56/58/59/66/68/73/82) [48]. HSV-1 was detected with PCR and Luminex xMAP technology, as decribed earlier [48].

#### HPyV detection with qPCR

SV40, JCV and BKV DNAs were detected by qPCR (Roche, Light Cycler 96, (Roche Diagnostics, Roche Molecular Diagnostics, Pleasanton, CA, USA) targeting their oncogenic large T antigen (T-ag) as decribed earlier ref [43]. TaqMan® Copy Number Reference Assay RNase was used as a reference gene (Applied Biosystems, 4403328). Table 5 summarizes the oligonucleotide sequences of the primers and probes used in qPCR as described by McNees et al. in 2005 [43]. The probes for the target genes SV40, JCV and BKV were labeled with 6-carboxyfluorescein (FAM), and for the reference gene RNase P, with 4,7,2′-trichloro-7′-phenyl-6-carboxyfluorescein (VIC). The oligonucleotides were provided by Applied Biosystems (Foster City, CA, USA). The primers were reconstituted in sterile water and the primers and probes were aliquoted and stored at −20°C in small volumes to minimize multiple uses of these reagents.

**Table 5:** Primers and probes for SV40, JCV and BKV detection with qPCR

Name	Sequence Detection
SV40 primer forward	GAT GGC ATT TCT TCT GAG CAA A
SV40 primer reverse	GAA TGG GAG CAG TGG TGG AA
JCV primer forward	TTC TTC ATG GCA AAA CAG GTC TT
JCV primer reverse	GAA TGG GAA TCC TGG TGG AA
BKV primer forward	CTT TCT TTT TTT TTT GGG TGG TGT T
BKV primer reverse	TTG CCA GTG ATG AAG AAG CAA
SV40 T-ag probe 5’-FAM	CAG GTT TTC CTC ATTAAA
JCV T-ag probe 5’ FAM	CCA CTT CTC ATT AAA TG
BKV T-ag probe 5' FAM	AGT GTT GAG AAT CTG C

Adapted from McNees et al., 2005 [41].

Using either strip tubes or 96-well plates (Roche LightCycler^®^ 96), and caps or adhesive covers of optical grade (Roche), 20 μl PCR reactions were prepared for SV40, JCV or BKV separately containing 900 nM of each primer, 100 nM of FAM-labeled probe, and 25μl of 2× TaqMan Universal PCR Master Mix (Applied Biosystems). For detection of the cellular gene, primer and probe components of the TaqMan RNAse P Control reagents (VIC dye) (Applied Biosystems) were used according to the manufacturer's instructions using the TaqMan Universal PCR Master Mix. Two microliters of standard plasmid dilutions or positive control samples or test DNA samples was added in duplicate outside the core facility after the tubes containing the master mix and negative controls were sealed. qPCR reaction conditions for amplification of all target genes were as follows: 50°C for 2min, denaturing at 95°C for 10min, and 45 cycles of denaturing at 95°C for 15s followed by annealing and extension at 60°C for 1min. Amplification data measured as an increase in reporter fluorescence were collected in real time and analyzed by the LightCycler® 96 Application and Instrument Software Sequence Detection (Roche Diagnostics, Indianapolis, US).

The linear standard curves for JCV and BKV were obtained with a serial dilution of plasmids ranging from 1.2^*^10^0 to 1.2^*^10^-2 ng/μl for JCV and 9.5^*^10^0 to 9.5^*^10^-3 ng/μl for BKV. COS1 cell line containing one copy of SV40 was used for SV40 standards using a dilution of DNA from 5.0^*^10^4 to 5.0^*^10^0 cells/μl, while the standards for the reference gene RNase P was acquired with a serial dilution of human placenta DNA extractions ranging from 5.09^*^10^2 to 5.09^*^10^-2 (Sigma-Aldrich, St. Louis, MO, USA).

The primers and probes used to detect the viral genes were specific for each virus and there was no cross reactivity within the dynamic range of the standard dilutions.

#### Multiplex PCR for HPyV detection using the Luminex platform

All the HPyV assays were performed as recently described by Sadeghi and coworkers [37]. In brief, 5 μl DNA template was combined with 20 μl multiplex reaction consisting of 12.5 μl of 2 x multiplex PCR mastermix (Qiagen), 0.2 μM of each forward primer and 1μM of each biotinylated reverse primer. The amplification conditions were 95°C for 15 min, 40 cycles at 94°C for 20 s, 50°C for 90 s, 71°C for 1 min and 20 s, and a final extension at 71°C for 10 min.

Luminex-based suspension array procedure (oligonucleotide coupling, hybridization, and measurement) were as described by in [30]. In brief, the 5’ amine C-12 modified oligonucleotide probes were coupled to a variety of carboxylated fluorescent microbeads (Luminex Corp., The Netherlands) according to the manufacturer's instructions (xMAP cookbook, Luminex). The probe-coupled beads were counted using a hemocytometer, and stored in the dark at +4°C. 45 μl of probe-bead mix combined with 5 μl of PCR products were hybridized for 40 minutes at 48°C, and incubated with streptavidin-phycoerythrin for 20 minutes at hybridization temperature. Measurement: after three washes, the signals of the beads and SAPE were measured in a Bio-Plex 200 (Bio-Rad).

### Statistical analysis

For categorical variables, the frequency tables were analyzed using the χ2-test, interpreted with the likelihood ratio (LR) or Fisher's exact statistics. Odds ratios (OR) and their 95% confidence intervals (95% CI) were calculated where appropriate, using the exact method. Differences in means of continuous variables were analyzed using Mann-Whitney's or Kruskal-Wallis's test for two and multiple independent samples, respectively. Univariate survival analysis for outcome measures (DSS) was based on Kaplan-Meier method, in which stratum-specific estimates were compared using the log-rank (Mantel-Cox) statistics. Only the patients treated with curative intent were taken into consideration in these survival calculations. All statistical tests were performed as two-sided and considered significant at p-value <0.05. Statistical analyses were run, using SPSS® 23.0.0.0 (SPSS, Inc., Chicago, USA) and STATA/SE 11.1 (STATA Corp., College Station, TX, USA) software packages.

## Abbreviations

DNA: deoxyribonucleic acid
DSS: disease specific survival
EBV: Ebstein Barr virus
FAM: 6-carboxyfluorescein
HNSCC: head and neck squamous cell carcinoma
HPV: human papillomavirus
HPyV: human polyomavirus
HSV: herpes simplex virus
LR: likelihood ratio
OR: odds ratios
pRb: retinoblastoma
PyV: polyomavirus
qPCR: quantitative polymerase chain reaction
SCC: squamous cell carcinoma
VIC: 4,7,2’-trichloro-7’-phenyl-6-carboxyfluorescein
